# Determinants of successful lifestyle change during a 6-month preconception lifestyle intervention in women with obesity and infertility

**DOI:** 10.1007/s00394-018-1798-7

**Published:** 2018-08-03

**Authors:** Matty D. A. Karsten, Anne M. van Oers, Henk Groen, Meike A. Q. Mutsaerts, Mireille N. M. van Poppel, Anouk Geelen, Cornelieke van de Beek, Rebecca C. Painter, Ben W. J. Mol, Tessa J. Roseboom, Annemieke Hoek, the LIFEstyle study group, J. M. Burggraaff, W. K. H. Kuchenbecker, D. A. M. Perquin, C. A. M. Koks, R. van Golde, E. M. Kaaijk, J. M. Schierbeek, G. J. E. Oosterhuis, F. J. Broekmans, N. E. A. Vogel, C. B. Lambalk, F. van der Veen, N. F. Klijn, P. E. A. M. Mercelina, Y. M. van Kasteren, A. W. Nap, R. J. A. B. Mulder, E. T. C. M. Gondrie, J. P. de Bruin

**Affiliations:** 1 Department of Obstetrics and Gynecology, University Medical Center Groningen, University of Groningen, Hanzeplein 1, 9713 GZ Groningen, The Netherlands; 2 Department of Obstetrics and Gynecology, Amsterdam UMC, University of Amsterdam, Meibergdreef 9, 1105 AZ Amsterdam, The Netherlands; 3 Department of Clinical Epidemiology, Biostatistics and Bioinformatics, Amsterdam UMC, University of Amsterdam, Meibergdreef 9, 1105 AZ Amsterdam, The Netherlands; 4 Department of Obstetrics and Gynecology, Medisch Spectrum Twente, Koningsplein 1, 7512 KZ Enschede, The Netherlands; 5 Department of Epidemiology, University Medical Center Groningen, University of Groningen, Hanzeplein 1, 9713 GZ Groningen, The Netherlands; 6 Department of Public and Occupational Health, Amsterdam Public Health Research Institute, Amsterdam UMC, VU University Amsterdam, Van der Boechorststraat 7, 1081 BT Amsterdam, The Netherlands; 7 Institute of Sport Science, University of Graz, Mozartgasse 14, 8010 Graz, Austria; 8 Division of Human Nutrition, Wageningen University and Research, Stippeneng 4, 6708 WE Wageningen, The Netherlands; 9 Department of Obstetrics and Gynecology, School of Medicine, Monash University, Clayton Road, Melbourne, Australia

**Keywords:** Lifestyle intervention, Obesity, Preconception, Determinants

## Abstract

**Purpose:**

To identify demographic, (bio)physical, behavioral, and psychological determinants of successful lifestyle change and program completion by performing a secondary analysis of the intervention arm of a randomized-controlled trial, investigating a preconception lifestyle intervention.

**Methods:**

The 6-month lifestyle intervention consisted of dietary counseling, physical activity, and behavioral modification, and was aimed at 5–10% weight loss. We operationalized successful lifestyle change as successful weight loss (≥ 5% weight/BMI ≤ 29 kg/m^2^), weight loss in kilograms, a reduction in energy intake, and an increase in physical activity during the intervention program. We performed logistic and mixed-effect regression analyses to identify baseline factors that were associated with successful change or program completion.

**Results:**

Women with higher external eating behavior scores had higher odds of successful weight loss (OR 1.10, 95% CI 1.05–1.16). Women with the previous dietetic support lost 0.94 kg less during the intervention period (95% CI 0.01–1.87 kg). Women with higher self-efficacy reduced energy intake more than women with lower self-efficacy (*p* < 0.01). Women with an older partner had an increased energy intake (6 kcal/year older, 95% CI 3–13). A high stage of change towards physical activity was associated with a higher number of daily steps (*p* = 0.03). A high stage of change towards weight loss was associated with completion of the intervention (*p* = 0.04).

**Conclusions:**

Determinants of lifestyle change and program completion were: higher external eating behavior, not having received previous dietetic support, high stage of change. This knowledge can be used to identify women likely to benefit from lifestyle interventions and develop new interventions for women requiring alternative support.

**Trial registration:**

The LIFEstyle study was registered at the Dutch trial registry (NTR 1530; http://www.trialregister.nl/trialreg/admin/rctview.asp?TC=1530).

**Electronic supplementary material:**

The online version of this article (10.1007/s00394-018-1798-7) contains supplementary material, which is available to authorized users.

## Background

In 2000, the World Health Organization declared obesity a pandemic and one of the most important current public health problems [1, 2]. Overweight and obesity are major risk factors for a number of chronic diseases, including diabetes, cardiovascular diseases, and cancer [3, 4]. In the Netherlands in 2012, 6% of women aged 20–29 years and 10% of those aged 30–39 years were obese [5]. In the United States, 37% of women of reproductive age were obese in 2013–2014 [6]. In women, obesity is associated with lower pregnancy rates, higher rates of obstetric complications [7–9], and it negatively affects maternal future health as well as health of the offspring [10].

To decrease the risks of obesity-related diseases, treatment consisting of lifestyle optimization, through a comprehensive program of lifestyle modification, is recommended [10–13]. However, lifestyle change has proven to be difficult and most lifestyle interventions have been shown to induce only modest changes in targeted behaviors [13–15]. Furthermore, non-completion rates (24%) of lifestyle intervention programs reduce treatment success [16, 17].

Nevertheless, the preconception period seems to be a time in which women are particularly receptive to advice about diet and lifestyle [18]. For example, studies have shown that interventions aimed at smoking cessation are more successful among women who intended to become pregnant [19, 20]. Potential beneficial effects on the health of a future child have been reported to be an important motivator for women to change their lifestyle [21].

Psychosocial and behavioral variables, such as higher motivation, higher self-efficacy, a more positive body-image, self-regulation skill use [22], fewer previous weight loss attempts [23, 24], and a higher socioeconomic status [25], have been linked to lifestyle change success. Further knowledge on determinants of success or failure to achieve lifestyle change through lifestyle intervention programs is important. Knowing what type of persons are successful in achieving their intended goals during an intervention program and being able to screen participants on certain characteristics before the start of an intervention program can assist in future intervention design and delivery of more individualized, and hopefully more effective interventions [13].

We conducted the LIFEstyle study, a randomized-controlled trial (RCT) including 577 women with obesity and infertility, which compared the effects of a 6-month preconception lifestyle intervention preceding infertility treatment to prompt infertility treatment [17]. The intervention was efficacious in the sense that it resulted in modest weight loss of 5.3 kg in women who completed the intervention. However, 22% of women did not complete the intervention [17]. The purpose of the current study was to investigate the determinants of successful lifestyle change and completion of the intervention program in women with obesity and infertility using data from women in the intervention arm of the RCT.

## Materials and methods

This study used data of the LIFEstyle study, which was a multicenter RCT in 577 women with obesity and infertility [body mass index (BMI) ≥ 29 kg/m^2^] aged between 18 and 39 years. In total, 290 women were allocated to the intervention group; one woman withdrew her informed consent. For the current study, we only used the data of women randomized to receive the intervention (*N* = 289). The design and main results of the LIFEstyle study have been reported previously [17, 26]. Infertility was defined as chronic anovulation or unsuccessful conception for at least 12 months [27]. Women with endocrinopathy, severe endometriosis, premature ovarian insufficiency, untreated preexisting hypertension, and women with a history of hypertension-related pregnancy complications were excluded [26].

### Lifestyle intervention

During the LIFEstyle study, women in the intervention group participated in a 6-month structured lifestyle intervention program, which was aimed at a weight loss of at least 5% of the original body weight. When the target weight reduction was met, or when BMI had decreased to below 29 kg/m^2^, or after finalization of the 6-month program, couples were eligible for infertility treatment. Infertility treatment was offered according to the Dutch guidelines for reproductive medicine and could consist of expectative management, ovulation induction, intrauterine inseminations, in vitro fertilization, or intracytoplasmic sperm injection depending on the diagnosis which the couple received after the infertility workup [28].

The lifestyle program consisted of a combination of dietary counseling, an increase in physical activity, and an individualized behavioral modification plan [11, 26, 29, 30]. Four individual consultations at the local hospital were planned in the first 3 months of the intervention and two additional sessions in the last 3 months. In between, four consultations by telephone or e-mail were scheduled. Trained intervention coaches, who had a degree in nursing or dietetics, guided participants throughout these consultations. Weight and height were measured by the intervention coach at baseline, and weight measurements were continued during the six individual consultations at the local hospital. During the four telephone/e-mail consultations in between, weight measures were provided by the women themselves. Coaches used a standardized software module to minimize practice variation. Information, including body measurements, energy intake, and physical activity, was captured in this system at all ten time points. Women were advised to consume a healthy diet with a caloric reduction of approximately 600 kilocalories (kcal) per day compared to their habitual energy intake, but a total energy intake of at least 1200 kcal/day. Besides the consultations, participants received feedback on food and energy intake on a daily basis using a web-based food diary ‘Eetmeter’ of the Netherlands Nutrition Center [31], which is linked to the Dutch food composition database [32]. This web-based food diary was used for counseling purposes and has not been validated for research purposes. Data on energy intake were collected by the web-based food diary during each consultation with the intervention coach and used to estimate mean energy intake per day in the period prior to each consultation. In addition, women were advised to be physically active with moderate intensity (60–85% of maximum heart rate frequency) for at least 30 min two-to-three times a week and to increase physical activity in daily life by taking 10,000 steps per day monitored by a pedometer (Yamax Digi-Walker SW 200, Develing International®, Bunschoten, The Netherlands). A diary was kept on these physical activities to establish self-monitoring. The pedometer was used to calculate mean daily steps during the intervention period prior to each consultation with the intervention coach. Changing behavior through motivational counseling was directed at: (1) awareness of actual lifestyle leading to overweight or obesity, (2) counseling healthy lifestyle measures: the benefit of a healthy lifestyle in relation to infertility, and spontaneous and treatment-dependent pregnancy chances, pregnancy complications, and perinatal outcome, and (3) formulating individualized goals embedded in a ‘patient contract’. During the intervention, individual goals were evaluated, participants received feedback from the intervention coaches, and goals were adapted if necessary.

### Outcome measures and determinants of outcomes

#### Lifestyle change outcomes

In this exploratory analysis, we aimed to identify determinants of lifestyle change and completion of the intervention program. We operationalized lifestyle change using the following dichotomous and continuous outcomes: successful weight loss (dichotomous), continuous weight loss in kilograms, change in daily energy intake (continuous), and change in daily number of steps (continuous) during the intervention program. Successful weight loss was defined as weight loss of at least 5% of original body weight or reaching a BMI ≤ 29.0 kg/m^2^ at any given moment during the 6-month intervention period, as this is in line with the aim of the intervention program. When a woman achieved the successful weight loss goal, she was allowed to stop the intervention program and was considered a completer of the intervention. Since the goal on an individual level was 5–10% weight loss and women formulated individual goals with the intervention coach prior to the intervention, she could also choose to continue losing weight (*N* = 28, 10%) within the 6-month intervention program.

Since weight loss is the result of the balance between energy intake and energy expenditure, we also analyzed the continuous variables ‘change in mean energy intake in kilocalories per day’, as measured by the web-based ‘Eetmeter’ [31] and ‘change in the mean number of steps per day’, as measured by the pedometer. All changes in steps and energy intake were calculated using the difference between baseline consultation and the last consultation during the intervention. When a woman became pregnant during the intervention period, subsequent continuous outcome measurements were censored from the longitudinal analysis.

#### Outcome completion of the intervention program

In addition, we investigated determinants of completion of the lifestyle intervention (dichotomous). Women were considered to have completed the intervention when they did not miss more than two consecutive coaching sessions and finalized the intervention at 6 months independent of the amount of weight loss. When a woman became pregnant or achieved successful weight loss before or within 6 months, and, therefore, did not reach the full 6-month length of the intervention period, the intervention was also considered completed.

#### Determinants of lifestyle change

Possible determinants of lifestyle change and data of questionnaires were all assessed at baseline and categorized into several domains, namely:

- *Demographic characteristics* Age, ethnicity, education level, socioeconomic status, and smoking of the woman were obtained from medical records. The socioeconomic status was based on the postal code using the status score from 2010, developed by the Netherlands Institute for Social Research [33]. This score reflects the social status of a neighborhood, compared to other neighborhoods in the Netherlands. A positive score represents a higher socioeconomic status, relative to the Dutch overall average of 0 (range − 5.27 to 2.15).
- *Infertility characteristics* duration of infertility in months, nulliparity, presence of anovulation, male factor infertility, or unexplained infertility were retrieved from medical records.
- *Anthropometric and weight characteristics* BMI in kg/m^2^, waist circumference in centimeter, and waist–hip ratio were measured by the intervention coach. Highest and lowest body weight over a period of the last 10 years, highest weight ever, weight variability in kg (calculated using the highest and lowest body weight during the past 10 years), and the number of the previous weight loss attempts during the last 5 years were all retrospectively questioned.
- *Metabolic characteristics* Degree of insulin resistance was quantified using the homeostasis model assessment of insulin resistance (HOMA-IR). This model was defined as fasting insulin concentration in µU/mL multiplied by fasting glucose concentration in mmol/L divided by 22.5 [34]. The presence of metabolic syndrome was identified using the 2001 revised National Cholesterol Education Program Adult Treatment Panel (rNCEP ATP III) criteria [35].
- *Psychosocial characteristics* Quality of Life (QoL) was assessed with the Short Form-36 (SF-36) questionnaire [36, 37], which measures overall physical and mental QoL.
  The stage of change was based on the transtheoretical model of Prochaska and DiClemente [38].
- This model describes the classification of participants into five stages of change; (1) precontemplation, (2) contemplation, (3) preparation, (4) action, and (5) maintenance. In our study, the stage of change for weight loss and physical activity behavior change were classified using two single-item questions. A higher score indicated a higher stage of change. The stage of change for weight loss [39] was derived from the following question: ‘Have you been trying to lose weight?’, with possible answer categories: (1) ‘No, and I am not planning to do so in the coming 6 months’, (2)‘No, but I am planning to do so in the coming 6 months’, (3)‘No, but I am planning to do so in the coming month’, (4)‘Yes, I have been trying to lose weight for less than 6 months’, and (5)’Yes, I have already been trying to lose weight for more than 6 months. The stage of change for physical activity [40, 41] was derived from the answer categories ranging from: (1) ‘At the moment I am not physically active on a regular basis and I do not intend to start in the near future’ (2) ‘At the moment I am not physically active on a regular basis, but I intend on becoming so in the near future’, (3) ‘During the past few months I occasionally engaged in exercise or sports’, (4) ‘During the last 6 months I engaged in exercise or sports at least 5 days a week for at least 30 min’, and (5) ‘During the past year I engaged in an intense workout at least three times a week’. The previous support in losing weight (either by dietician or partner) or receiving no support was derived asking whether the following single-item statements were applicable: ‘I received help from a dietician’, ‘I received help from my partner’, or ‘I did not receive any support’. Self-efficacy was assessed, using the non-validated single-item statement ‘I think I’ll manage to reach my weight goal, when I’m trying to lose weight’ and body satisfaction, using the non-validated single-item statement ‘I am satisfied with my own body weight’, both were scored on a five-point Likert scale. We assessed three subscales of the Dutch Eating Behavior Questionnaire (DEBQ) [42]: emotional eating, external eating, and restrained eating. Emotional eating is described as the eating in response to emotional arousal states such as fear, anger, or anxiety. External eating: eating in response to external food cues such as sight and smell of food. Restraint eating: overeating after a period of slimming when the cognitive resolve to diet is abandoned [42].

- *Diet and physical activity characteristics*: Mean energy intake in kcal per day was estimated using a web-based food diary of the Netherlands Nutrition Center and mean steps per day were monitored using a pedometer. Meeting the recommendations for fruit and vegetable intake was based on the Dutch Guidelines for a healthy diet 2006 [43], which advises a minimal recommended fruit and vegetable intake of 200 g per day of which a maximum of 100 g of fruit could be substituted by 1 glass of fruit juice. Dietary intake was assessed using a self-administered Food Frequency Questionnaire (FFQ). The first part of the FFQ was obtained from the standardized questions on food consumption used for the Public Health Monitor in the Netherlands [44]. This standardized questionnaire consisted of questions about type of cooking fats, type of bread, frequency of breakfast use, frequency of consumption, and portion size of vegetables, fruits, and fruit juice. This first part has been supplemented with a second part, consisting of additional frequency and portion size questions about snack intake, the usage of sugar containing and alcoholic beverages, and the use of creamer and/or sugar in coffee and tea. Frequency of consumption was asked per week (breakfast, vegetable, and fruit) or per month (sugar containing and alcoholic beverages, and snacks). We were not able to estimate energy intake from the FFQ, since it only determined food groups. Therefore, it was used to determine the intake of specific food groups and dietary behaviors as potential determinants of successful lifestyle change. The FFQ was collected at baseline, 3, 6, and 12 months after randomization. For this analysis, the FFQ at baseline was used.
- To assess the total amount of moderate to vigorous physical activity and if participants met the Dutch guidelines for physical activity, the validated Short Questionnaire to Assess Health-enhancing physical activity questionnaire (SQUASH) was used [45]. Based on the Ainsworth’s compendium of physical activities [46], activities were subdivided into 2 to < 4 Metabolic Equivalent of Task (MET, light), 4-<6.5 MET (moderate), and ≥ 6.5 MET (vigorous). These cut-off points were chosen based on the Dutch physical activity guideline [47].

- *Partner characteristics* Age, BMI, ethnicity, and smoking behavior of the partner were based on medical records.

### Statistical analyses

Descriptive statistics are given as *n*, %, median, and interquartile range (IQR) where appropriate. We examined the data regarding determinants and outcomes for plausibility, excluding outliers from further analyses. We omitted outliers in mean energy intake (web-based food diary ‘Eetmeter’ < 500 and > 5000 kcal per day, *N* = 26) and steps (pedometer > 40,000 steps per day, *N* = 6). In addition, all continuous variables used as determinants were screened for outliers and improbable values were omitted.

Univariable logistic regression analyses were performed to identify determinants of lifestyle change of the dichotomous outcomes. The results of the logistic regression analyses are reported as the odds ratio (OR) with the corresponding 95% confidence interval (CI). Mixed-effect regression analysis was performed to identify determinants of lifestyle change on the repeated measurements of the continuous outcomes (weight, energy intake, and number of steps). Mixed-effect regression analysis handles non-independent data, such as repeated measurements, and in contrast to ANOVA analysis, it is not limited to complete-case analysis. We performed baseline correction by introducing the baseline measurement of the dependent variable as a covariate in the mixed-effect regression models, and we included a random intercept. Since pregnancy is known to affect body weight and energy intake, we censored measurements on weight, energy intake, and number of steps of women with an ongoing pregnancy from the conception date onwards. The results of the mixed-effect regression models are reported as regression coefficients (*β*) and the corresponding 95% CI, and indicate mean change throughout the intervention period.

To identify independent determinants of lifestyle change or completion of the intervention program, all determinants with a *p* value < 0.05 were entered in the multivariable binary logistic regression model or multivariable mixed-effect regression model. When the overall *p* value for a categorical variable was not significant, but one of the subcategories was, the entire categorical variable was considered as not being statistically significant. Since our analyses are exploratory, we did not adjust for multiple testing [48]. All analyses were performed using IBM SPSS Statistics (version 22.0; SPSS Inc, Chicago, IL, USA).

## Results

Baseline characteristics of women in the intervention group are shown in Table 1.

**Table 1:** Baseline characteristics of 289 women randomized to the intervention group of the LIFEstyle RCT

	*N*	LIFEstyle Intervention Program
Age of women (years; mean ± SD)	289	29.7 ± 4.5
Non-Caucasian (yes; *N*, %)	289	33 (11.4)
Education level (*N*, %)	276	
No education/primary school		17 (6.2)
Secondary education		66 (23.9)
Intermediate vocational education		135 (48.9)
Higher vocational education and university		58 (21.0)
Socioeconomic status score^a^ (median, IQR)	230	− 0.53 (− 1.24 to 0.38)
Current smoker (yes; *N*, %)	285	76 (26.3)
Baseline BMI (kg/m^2^; median; IQR)	288	36.1 (33.4–38.6)
Baseline weight (kg; mean ± SD)	288	103.5 ± 13.9
Baseline energy intake (kcal/day; median; IQR)	213	1890 (1600–2200)
Baseline steps (steps/day; median; IQR)	230	6000 (4000–8000)
Age of partner (years; mean ± SD)	289	33.5 ± 6.1
Smoking partner (yes; *N*, %)	286	117 (40.9)
Baseline BMI of partner (kg/m^2^; median, IQR)	247	27.7 (24.4–31.0)

*RCT* randomized-controlled trial, *BMI* body mass index, *IQR* interquartile range

^a^Socioeconomic status score [Netherlands Institute for Social Research (SCP)] in 2010 relative to Dutch average of 0; a higher score represents a higher socioeconomic status

### Determinants of successful weight loss

Loss of ≥ 5% of the original body weight or reaching a BMI ≤ 29 kg/m^2^ was achieved by 119/289 women (41%) within 6 months after randomization, of which 116 women reached ≥ 5% weight loss and 18 women reached a BMI ≤ 29 kg/m^2^ (not mutually exclusive). A lower BMI, a lower waist circumference, a lower highest weight in the past 10 years, not receiving the previous support in losing weight by a dietician, and a higher score on external eating behavior were significantly associated with successful weight loss in the univariable logistic regression analyses (Additional Table S1) and were, therefore, included in the multivariable model (Table 2).

**Table 2:** Determinants of successful weight loss in 289 women randomized to the intervention group

Determinants	OR (95% CI) univariable	*p*	OR (95% CI) multivariable	*p*
BMI (kg/m^2^)	0.87 (0.81–0.94)	**0.001**	0.93 (0.81–1.07)	0.29
Waist circumference (cm)	0.97 (0.95–1.00)	**0.02**	1.00 (0.96–1.04)	0.87
Highest weight past 10 years (kg)	0.98 (0.96–0.99)	< **0.01**	0.98 (0.96–1.01)	0.28
Previous support by a dietician	0.56 (0.34–0.98)	**0.04**	0.53 (0.29–1.00)	0.05
External eating (units in DEBQ)	1.07 (1.02–1.12)	< **0.01**	1.10 (1.05–1.16)	< **0.01**

Bold value indicates a statistically significant difference with a *p*-value less than 0.05

Results of uni- and multivariable logistic regression analyses on determinants of successful weight loss. To identify independent determinants of lifestyle change or completion of the intervention program, all determinants with a *p* value < 0.05 were entered in the multivariable binary logistic regression model. ORs indicate the odds of successful weight loss given that the determinant is present. Successful weight loss is defined as loss of ≥ 5% of original body weight or reaching a BMI ≤ 29 kg/m^2^. Full univariable logistic regression results are provided in Additional Table S1.

*OR* odds ratio, *BMI* body mass index, *DEBQ* Dutch Eating Behavior Questionnaire

The multivariable logistic regression model showed that women with higher scores on external eating behavior had higher odds of successful weight loss (OR 1.10, 95% CI 1.05–1.16).

### Determinants of weight loss (as continuous variable)

Mean weight loss was 5.20 kg (95% CI − 5.72 to − 4.68) at 6 months after randomization compared to the baseline visit (*p* < 0.001). A higher BMI, a longer duration of infertility, a higher number of past weight loss attempts, a higher stage of change towards weight loss, and receiving the previous support by a dietician were significantly associated with the magnitude of weight loss in the univariable mixed-effect regression model (Additional Table S2) and were, therefore, included in the multivariable mixed-effect regression model (Table 3).

**Table 3:** Determinants of weight loss in kilograms in 289 women randomized to the intervention group

Determinants	*β* kg (95% CI) univariable	*p*	*β* kg (95% CI) multivariable	*p*
BMI (kg/m^2^)	0.25 (0.08–0.42)	< **0.01**	0.07 (− 0.14 to 0.28)	0.51
Duration of infertility (months)	0.02 (0.00–0.03)	**0.03**	0.02 (− 0.00 to 0.04)	0.08
Number of weight loss attempts in past 5 years	n.a.	< **0.01**	n.a.	0.08
None	[ref]	[ref]	n.a.	
1 Attempt	0.80 (− 1.09 to 2.69)	0.40	[ref]	[ref]
2–3 Attempts	0.47 (− 1.28 to 2.22)	0.60	− 0.83 (− 2.25 to 0.59)	0.25
4–5 Attempts	0.76 (− 1.10 to 2.62)	0.42	− 0.87 (− 2.45 to 0.71)	0.28
> 5 Attempts	2.26 (0.57–3.95)	< **0.01**	0.42 (− 0.98 to 1.82)	0.56
Stage of change: weight loss	n.a.	**0.001**	n.a.	0.15
Precontemplation	n.a.	n.a.	n.a.	n.a
Contemplation	− 2.21 (− 6.73 to 2.302)	0.34	− 1.84 (− 6.41 to 2.73)	0.43
Preparation	− 1.11 (− 2.35 to 0.13)	0.08	− 0.16 (− 1.55 to 1.24)	0.83
Action	− 1.90 (− 2.80 to − 0.99)	< 0.001	− 1.12 (− 2.15 to − 0.09)	0.03
Maintenance	[ref]	[ref]	[ref]	[ref]
Previous support by a dietician	1.37 (0.48–2.27)	< **0.01**	0.94 (0.01–1.87)	**0.047**

Bold value indicates a statistically significant difference with a *p*-value less than 0.05

Results of uni- and multivariable mixed-effect regressions models on determinants of weight loss. With correction for baseline BMI and including a random intercept. Mixed-effect regression analysis was performed to identify determinants of lifestyle change on the repeated measurements of the continuous weight loss outcome. All determinants with a *p* value < 0.05 were entered in the multivariable model. We censored weight measurements of women with an ongoing pregnancy from the conception date onwards. The results of the mixed-effect regression models are reported as regression coefficients (*β*) and the corresponding 95% CI, and indicate mean change throughout the intervention period. A negative number indicates additional weight loss; a positive number indicates less weight loss. Full univariable mixed-effect regression results are provided in Additional Table S2. The stage of change for weight loss was derived from the following question: ‘Have you been trying to lose weight?’, with possible answer categories: (1) Precontemplation: ‘No, and I am not planning to do so in the coming 6 months’, (2) Contemplation: ‘No, but I am planning to do so in the coming 6 months’, (3) Preparation: ‘No, but I am planning to do so in the coming month’, (4) Action: ‘Yes, I have been trying to lose weight for less than 6 months’ and (5) Maintenance: ‘Yes, I have already been trying to lose weight for more than 6 months’

*β* regression coefficient, *CI* confidence interval, *BMI* body mass index, *n.a*. not applicable

In the multivariable mixed-effect regression model, receiving the previous support by a dietician to lose weight was the only independent determinant of continuous weight loss. Women who had the previous support by a dietician lost 0.94 kg less weight during the intervention period (95% CI 0.01–1.87 kg) than women who did not receive support (Table 3).

### Determinants of energy intake

During the intervention, women reduced their mean energy intake per day by 472 kilocalories (95% CI − 536 to − 409) at 6 months after randomization compared to the baseline visit (*p* < 0.001). Self-efficacy and a higher age of the partner were significantly associated with changes in energy intake in the univariable mixed-effect regression model (Additional Table S2), and were, therefore, included in the multivariable mixed-effect regression model (Table 4).

**Table 4:** Determinants of energy intake and number of steps in 289 women randomized to the intervention group

Determinants of energy intake	*β* (95% CI) per 100 kcal Univariable	*p*	*β* (95% CI) per 100 kcal Multivariable	*p*
Self-efficacy	n.a.	< **0.01**	n.a.	< **0.01**
Extremely unlikely	[ref]	[ref]	[ref]	[ref]
Unlikely	− 3.31 (− 7.48 to 0.87)	0.12	− 3.14 (− 7.20 to 0.92)	0.13
Do not know/neutral	− 2.75 (− 6.65 to 1.15)	0.17	− 1.87 (− 5.71 to 1.96)	0.34
Likely	− 2.41 (− 6.31 to 1.50)	0.23	− 1.58 (− 5.42 to 2.26)	0.42
Extremely likely	− 3.75 (− 7.69 to 0.19)	0.06	− 2.89 (− 6.76 to 0.98)	0.14
Age of partner (years)	0.06 (0.01–0.11)	**0.01**	0.08 (0.03–0.13)	< **0.01**

Determinants of number of steps	*β* (95% CI) per 1000 steps Univariable	*p*	*β* (95% CI) per 1000 steps Multivariable	*p*
Stage of change: physical activity	n.a.	**0.04**	n.a.	**0.03**
Precontemplation	− 0.18 (− 2.37 to 2.00)	0.87	− 0.97 (− 3.13 to 1.20)	0.38
Contemplation	− 0.35 (− 1.45 to 0.76)	0.53	− 0.62 (− 1.68 to 0.44)	0.25
Preparation	0.03 (− 1.04 to 1.11)	0.95	0.03 (− 1.02 to 1.08)	0.95
Action	1.23 (0.09–2.37)	**0.04**	0.99 (− 0.11 to 2.09)	0.08
Maintenance	[ref]	[ref]	[ref]	[ref]
Insulin resistance	0.19 (0.02–0.37)	**0.03**	0.12 (− 0.04 to 0.28)	0.15
Metabolic syndrome	1.05 (0.32–1.78)	< **0.01**	0.56 (− 0.16 to 1.28)	0.13

Bold value indicates a statistically significant difference with a *p*-value less than 0.05

Results of uni- and multivariable mixed-effect regressions models on determinants of energy intake and the number of steps. With correction for baseline energy intake or steps and including random intercept. Mixed-effect regression analysis was performed to identify determinants of lifestyle change on the repeated measurements of the continuous outcomes on energy intake and number of steps. All determinants with a *p* value < 0.05 were entered in the multivariable model. Energy intake estimates and step measurements of women with an ongoing pregnancy were censored from the conception date onwards. The results of the mixed-effect regression models are reported as regression coefficients (*β*) and the corresponding 95% CI and indicate mean change throughout the intervention period. A negative number indicates a decrease in the intake of kcal/steps; a positive number indicates an increase the intake of kcal/steps. Full univariable mixed-effect regression results are provided in Additional Table S2. Self-efficacy: using the single self-administered question ‘I think I’ll manage to reach my weight goal, when I’m trying to lose weight’ was scored on a five-point Likert scale ranging from ‘extremely unlikely’ to ‘extremely likely’. The stage of change for physical activity was derived from the answer categories ranging from: (1) precontemplation: ‘At the moment I am not physically active on a regular basis and I do not intend to start in the near future’ (2) contemplation: ‘At the moment I am not physically active on a regular basis, but I intend on becoming so in the near future’, (3) preparation: ‘During the past few months I occasionally engaged in exercise or sports’, (4) action: ‘During the last 6 months I engaged in exercise or sports at least 5 days a week for at least 30 min’, and (5) maintenance: ‘During the past year, I engaged in an intense workout at least three times a week’

*n.a*. not applicable, *β* regression coefficient, *CI* confidence interval

In the multivariable mixed-effect regression model, both a higher age of the partner and self-efficacy remained independently associated with energy intake. For each year increase in the age of the partner, the daily energy intake increased by 6 kcal (95% CI 3–13). Furthermore, self-efficacy was significantly associated with energy intake (*p* < 0.01), i.e., women with the highest self-efficacy on the Likert scale had decreased mean energy intake relative to women with the lowest self-efficacy level (*p* < 0.01).

### Determinants of number of steps

Women increased their mean number of steps per day by 3231 steps (95% CI 2540–3921) at 6 months after randomization compared to the baseline visit (*p* < 0.001). A higher degree of insulin resistance, presence of metabolic syndrome, and a higher stage of change towards physical activity were significantly associated with the daily number of steps in the univariable mixed-effect regression model (Additional Table S2) and were, therefore, included in the multivariable mixed-effect regression model (Table 4).

In the multivariable model, the stage of change towards physical activity was significantly associated with the daily number of steps (*p* = 0.03), with a trend towards more steps in women who were increasingly ready to change, compared to women in the maintenance stage of change.

### Determinants of completion of the lifestyle intervention

In total, 226 (78%) women completed the lifestyle intervention. Reasons for discontinuing the intervention program were: a lack of motivation (*n* = 40), relationship problems with their partner (*n* = 12), and other reasons (*N* = 11). In total, 44 women (15%) had an ongoing pregnancy (> 10 weeks) during the intervention period. Current smoking, nulliparity, stage of change towards weight loss, a higher restrained eating behavior score, and a higher BMI of the partner were significantly associated with completion of the intervention program in the univariable logistic regression analyses (Additional Table S3) and were, therefore, included in the multivariable model (Table 5).

**Table 5:** Determinants of completion of the lifestyle intervention program in 289 women randomized to the intervention group

Determinants	OR (95% CI) univariable	*p*	OR (95% CI) multivariable	*p*
Current smoker	0.55 (0.30–0.99)	**0.047**	1.70 (0.76–3.76)	0.19
Nulliparous	2.02 (1.03–3.93)	**0.04**	1.07 (0.99–1.16)	0.08
Stage of change: weight loss	n.a	**0.01**	n.a.	**0.04**
Precontemplation	n.a.	n.a.	n.a.	n.a.
Contemplation	n.a.	n.a.	n.a.	n.a.
Preparation	0.43 (0.19–1.00)	**0.049**	0.40 (0.15–1.09)	0.07
Action	1.93 (0.90–4.15)	0.09	1.78 (0.74–4.26)	0.20
Maintenance	[ref]	[ref]	[ref]	[ref]
Restrained eating (DEBQ)	1.07 (1.02–1.12)	**0.01**	1.05 (0.98–1.12)	0.14
BMI of partner	1.07 (1.00–1.14)	**0.04**	1.07 (0.99–1.16)	0.08

Bold value indicates a statistically significant difference with a *p*-value less than 0.05

Results of uni- and multivariable logistic regression analyses on determinants of completion. To identify independent determinants of lifestyle change or completion of the intervention program, all determinants with a *p* value < 0.05 were entered in the multivariable binary logistic regression model. Full univariable logistic regression results are provided in Additional Table S3. ORs indicate odds for completion of the lifestyle intervention program as defined by not missing ≥ 2 consecutive sessions given that the determinant is present

*DEBQ* Dutch Eating Behavior Questionnaire, *BMI* body mass index, *n.a*. not applicable, *CI* confidence interval

The multivariable logistic regression model showed that only the stage of change towards weight loss was independently associated with the odds on completing the lifestyle intervention (*p* = 0.04), with women in the preparation phase having a higher odds of completing the intervention program, compared to women in the maintenance phase.

## Discussion

In women with obesity and infertility who participated in a 6-month lifestyle intervention program, a higher score on external eating behavior was independently associated with successful weight loss of ≥ 5% of original body weight or achieving a BMI ≤ 29 kg/m^2^. In addition, women without a history of the previous support by a dietician lost more weight than women who did have such a history.

External eating is associated with impulsiveness and lower self-discipline [49, 50]. Since our intervention included specific advice about what to do in situations that would trigger women to eat, this may have helped external eaters more in changing their lifestyle than women with other types of eating behaviors. Furthermore, since the intervention specifically focused on behavior change, also in situations that would usually be associated with unhealthy eating (such as smelling or seeing food at display), this may have been most helpful to external eaters (as it prepared them for difficult situations) and less so for other types of women such as emotional eaters. In our study, we did not find an association between emotional eating and weight loss success.

Women who had the previous support from a dietician lost less weight during the intervention, compared to women without prior support. It is possible that some women in our study represent a selection of women failed to lose weight after having been counseled previously about healthy lifestyle options. They could, therefore, be less susceptible to a repetition of the support offered during our lifestyle intervention program. Possibly, these women may have other underlying causes for their obesity, such as low self-esteem, lack of motivation or the previous trauma and, therefore, need different types of support [51, 52]. The finding that the previous counseling by a dietician has negative effects on weight loss is in line with the existing literature, showing that fewer previous weight loss attempts and less previous dieting are predictors of successful weight loss [24].

Many trials and observational cohorts have reported that a higher baseline BMI is associated with greater (initial) weight loss or weight loss maintenance over time [53–55]. However, reviews of BMI trajectories of weight loss [56] and BMI classes [57] reported no associations between those with an initial loss trajectory or higher BMI classes and weight loss outcomes. This is in line with our findings that baseline BMI was not an independent predictor of weight loss success.

Women with older partners increased their energy intake more than women with younger partners; however, the effect size was small, 8 kcal increase in energy intake for every year increase in age of the partner. It is difficult to place this finding into context, although it is known that obesity, dietary, and general health behaviors tend to cluster between spouses and within families [58, 59]. The magnitude of spousal concordance may differ per age group, since couples are usually close in age. In the literature, the proportion of both spouses reporting physical inactivity was highest in the oldest age group [60]. We, however, found no literature on the correlation of (dietary) habits in couples of whom one of the spouses was older.

Women with a higher level of self-efficacy had decreased energy intake relative to women with the lowest self-efficacy level. This finding is in agreement with literature, suggesting that high self-regulation skills and high self-efficacy are associated with a reduction in energy intake and increased physical activity [22].

Increased stage of change towards physical activity was significantly associated with increased daily number of steps. Women in the action phase of stage of change towards physical activity were more likely to increase their number of steps compared to women in the maintenance phase. Furthermore, stage of change towards weight loss also increased the odds of completing the lifestyle intervention. These results are in line with a review in which motivational readiness was found to be positively associated with physical activity [22]. Thus, it is important to assess the stage of change and incorporate motivational counseling in consultations with health care providers and lifestyle interventions [61]. Surprisingly, women in the action phase of stage of change for physical activity were more likely to increase their number of steps compared to women in the maintenance phase. The formulations for action and maintenance phase of the stage of change towards physical activity used in our study differed in the mentioned frequency and intensity, which might explain this unexpected finding.

A major strength of our study is that it is the first study evaluating the determinants of lifestyle change among women with obesity and infertility. The participants were of reproductive age and were, except for their obesity, generally in good health. The previous studies mainly focused on older obese patients with obesity-related comorbidities, such as hypertension or diabetes [62–64] and, therefore, our study fills a gap in literature. Another strength is the prospective design. All determinants were collected at baseline, at the start of the trial. Furthermore, using data of the intervention arm of an RCT, the possibility of allocation bias, often found in observational studies of lifestyle interventions, was eliminated. We used a robust statistical method, mixed-effect regression models, to analyze the continuous longitudinal outcomes. This method takes the within person dependence of the data into account and does not rely on complete-case data, so we were able to use all available data points.

Limitations of our study should be noted. Although we used several validated questionnaires, some of the determinants which we investigated originated from single-item questions. Therefore, some constructs that were investigated in our study, such as self-efficacy, may have limited validity. Furthermore, our RCT was not set up for analyses of determinants of lifestyle change within the intervention group and, therefore, type II errors might have occurred. We measured energy intake with a web-based food diary (‘Eetmeter’). This food diary was used for feasibility reasons, because it is online available throughout the Netherlands, but has not been not validated for research purposes. Furthermore, it is known that self-reported energy intake is subject to bias in general and even more in obese women who are known to under-report their intake of unhealthy foods [65, 66]. Due to these shortcomings, the ‘Eetmeter’ may, therefore, not have been able to completely identify determinants of energy intake. We did not find any consistent determinants across the domains assessed. This could be a reflection of the exploratory nature of the study. Our results are, however, in line with a comprehensive review of Teixeira et al. [22], where no consistent mediators for dietary intake in the long term (> 12 months) were found.

In our study, the determinants self-efficacy and body satisfaction were derived from non-validated single-item statements. In spite of this, self-efficacy predicted successful lifestyle change: higher self-efficacy was associated with a lower energy intake. However, a single-item question could be as predictive as a validated 20-item scale [67]. Thus, the use of a single-item statement may, therefore, be sufficient to show an association with lifestyle success.

A large body of evidence exists on the association between lower socioeconomic status, lower education level, and an increased risk of becoming overweight and obese [68–71]. In our study population, neither low educational level nor low socioeconomic status was identified as a determinant of lifestyle change. This suggests that the lifestyle intervention program is equally effective for women of lower and higher socioeconomic status.

## Conclusions

The determinants of lifestyle change and completion of the intervention in women with obesity and infertility were: higher external eating behavior, not having received the previous dietetic support, a higher self-efficacy level, higher age of the partner, and a high stage of change.

Knowledge of the determinants of success within lifestyle interventions is important, since this knowledge can help to identify people at risk of suboptimal results and can help to develop new interventions for women who require alternative support. Intervention programs should be tailored towards women with different types of eating behaviors, self-efficacy levels, stages of change, and whether women received the previous help from a dietician. Our type of intervention was suitable for women with external eating behavior. Women who experienced emotional eating may benefit from in-depth psychological help during the intervention program. Stages of change can be used as a proxy to whether women are ready for behavior change. Interventions should, therefore, be targeted towards women with higher stages of change or enhance the stage of change prior to the intervention.

## Electronic supplementary material

Below is the link to the electronic supplementary material.

Supplementary material 1 (DOCX 144 KB)
