# Supplementary material for: Determinants of successful lifestyle change during a 6-month preconception lifestyle intervention in women with obesity and infertility
Source: Eur J Nutr. 2018 Aug 3;58(6):2463–75. doi: 10.1007/s00394-018-1798-7 (PMC6689274; doi:10.1007/s00394-018-1798-7)
Supplement: Supplementary file 1 — Supplementary material 1 (DOCX 144 KB) [file 394_2018_1798_MOESM1_ESM.docx]

# Additional Table S1. Determinants of successful weight loss

| **Category** | **Determinant** | | | | | | **N** | **Successful weight loss**  **(median, IQR or N, %)** | **Unsuccessful weight loss**  **(median, IQR or N, %)** | **Odds ratio (95% CI)** | ***p*** |
| --- | --- | --- | --- | --- | --- | --- | --- | --- | --- | --- | --- |
| *Demographic characteristics* | Age of women (years) | | | | | | 261 | 29.9 (26.7 – 33.5) | 29.7 (26.1 – 32.8) | 1.02 (0.96 – 1.07) | 0.58 |
|  | Non-Caucasian | | | | | | 261 | 14 / 105 (11.8) | 15 / 127 (10.6) | 1.13 (0.52 – 2.45) | 0.76 |
|  | Education level | | | | | | 250 |  |  | 1.29 (0.93 – 1.77) | 0.12 |
|  |  | | | | | no education / primary school |  | 5 / 116 (4.3) | 6 / 134 (4.5) | *[ref]* | *[ref]* |
|  |  | | | | | secondary education |  | 24 / 116 (20.7) | 39 / 134 (29.1) | 0.74 (0.20 – 2.69) | 0.65 |
|  |  | | | | | intermediate vocational education |  | 57 / 116 (49.1) | 64 / 134 (47.8) | 1.07 (0.31 – 3.69) | 0.92 |
|  |  | | | | | higher vocational education and university |  | 30 / 116 (25.9) | 24 / 134 (18.7) | 1.44 (0.39 – 5.28) | 0.58 |
|  | Socioeconomic status score ^1^ | | | | | | 208 | -0.34 (-1.04 – 0.54) | -0.70 (-1.39 – 0.17) | 1.25 (0.99 – 1.59) | 0.07 |
|  | Current smoker | | | | | | 258 | 30 / 118 (25.4) | 36 / 140 (25.7) | 0.99 (0.56 – 1.73) | 0.99 |
|  |  | | | | | |  |  |  |  |  |
| *Infertility characteristics* | Duration of infertility (months) | | | | | | 261 | 23.0 (15.0 – 35.0) | 21.5 (14.0 – 41.3) | 1.00 (0.99 – 1.01) | 0.67 |
|  | Nulliparous | | | | | | 249 | 90 / 111 (81.1) | 114 / 138 (82.6) | 0.90 (0.47 – 1.72) | 0.76 |
|  | Anovulatory | | | | | | 260 | 49 / 118 (41.5) | 66 / 142 (46.5) | 0.81 (0.50 – 1.34) | 0.42 |
|  | Male factor infertility | | | | | | 260 | 31 / 118 (26.3) | 30 / 142 (21.1) | 1.33 (0.75 – 2.36) | 0.33 |
|  | Unexplained infertility | | | | | | 260 | 36 / 118 (30.5) | 43 / 142 (30.3) | 1.01 (0.60 – 1.72) | 0.97 |
|  |  | | | | | |  |  |  |  |  |
| *Anthropometric and weight characteristics* | BMI (kg/m^2^) | | | | | | 261 | 35.3 (32.8 – 37.3) | 37.0 (34.7 – 39.0) | 0.87 (0.81 – 0.94) | **0.001** |
|  | Waist circumference (cm) | | | | | | 257 | 106.0 (100.0 – 112.0) | 109.0 (102.0 – 115.0) | 0.97 (0.95 – 1.00) | **0.02** |
|  | Waist/hip ratio | | | | | | 255 | 0.86 (0.82 – 0.90) | 0.86 (0.82 – 0.91) | 0.97 (0.66 – 1.41) ^a^ | 0.86 |
|  | Highest weight in past 10 years (kg) | | | | | | 229 | 106.0 (98.0 – 115.0) | 112.0 (100.0 – 120.0) | 0.98 (0.96 – 0.99) | **<0.01** |
|  | Highest weight ever | | | | | | 219 |  |  |  | 0.13 |
|  |  | same as current weight | | | | |  | 28 / 104 (26.9) | 26 / 115 (22.6) | *[ref]* | *[ref]* |
|  |  | 1 – 5 kg above current weight | | | | |  | 42 / 104 (40.4) | 36 / 115 (31.3) | 1.08 (0.54 – 2.17) | 0.82 |
|  |  | 6 – 10kg above current weight | | | | |  | 19 / 104 (18.3) | 22 / 115 (19.1) | 0.80 (0.36 – 1.18) | 0.60 |
|  |  | > 10 kg above current weight | | | | |  | 15 / 104 (14.4) | 31 / 115 (27.0) | 0.45 (0.20– 1.02) | 0.05 |
|  | Weight variability in past 10 years ^3^ (kg) | | | | | | 223 | 29.0 (20.5 – 38.5) | 31.0 (22.0 – 40.0) | 0.99 (0.97 – 1.01) | 0.43 |
|  | Number of weight loss attempts in past 5 years | | | | | | 232 |  |  |  | 0.77 |
|  |  | | None | | | |  | 9 / 111 (8.1) | 7 / 121 (5.8) | *[ref]* | *[ref]* |
|  |  | | 1 attempt | | | |  | 17 / 111 (15.3) | 17 / 121 (14.0) | 0.78 (0.24 – 2.57) | 0.68 |
|  |  | | 2 – 3 attempts | | | |  | 32 / 111 (28.8) | 29 / 121 (24.0) | 0.86 (0.28 – 2.60) | 0.79 |
|  |  | | 4 – 5 attempts | | | |  | 16 / 111 (14 .4) | 20 / 121 (16.5) | 0.62 (0.19 – 2.04) | 0.43 |
|  |  | | > 5 attempts | | | |  | 37 / 111 (33.3) | 48 / 121 (39.7) | 0.60 (0.20 – 1.76) | 0.35 |
|  |  | | | | | |  |  |  |  |  |
| *Metabolic characteristics* | Insulin resistance (HOMA-IR) | | | | | | 228 | 2.9 (1.9 – 3.9) | 3.0 (2.2 – 4.3) | 0.93 (0.81 – 1.06) | 0.27 |
|  | Metabolic syndrome | | | | | | 220 | 51 / 103 (49.5) | 64 / 117 (54.7) | 0.81 (0.48 – 1.38) | 0.44 |
|  |  | | | | | |  |  |  |  |  |
| *Psychosocial characteristics* | Quality of Life^4^ | | | | | |  |  |  |  |  |
|  |  | | | physical component (score) | | | 218 | 53.0 (47.9 – 55.3) | 51.6 (45.2– 55.8) | - 1. (0.98 – 1.04) | 0.42 |
|  |  | | | mental component (score) | | | 218 | 53.0 (46.8 – 56.0) | 52.5 (45.4 – 55.4) | 1.01 (0.98 – 1.04) | 0.47 |
|  | Readiness to change: weight loss | | | | | | 228 |  |  |  | 0.51 |
|  |  | | | precontemplation | | |  | 0 / 109 (0.0) | 0 / 119 (0.0) | *n.a.* |  |
|  |  | | | contemplation | | |  | 1 / 109 (0.9) | 1 / 119 (0.8) | 1.42 (0.09 – 23.40) | 0.62 |
|  |  | | | preparation | | |  | 19 / 109 (17.4) | 17 / 119 (14.3) | 1.58 (0.73 – 3.46) | 0.25 |
|  |  | | | action | | |  | 53 / 109 (48.6) | 50 / 119 (42.0) | 1.50 (0.84 – 2.67) | 0.17 |
|  |  | | | maintenance | | |  | 36 / 109 (33.0) | 51 / 119 (42.9) | [ref] | *[ref]* |
|  | Readiness to change: physical activity | | | | | | 229 |  |  |  | 0.65 |
|  |  | | | precontemplation | | |  | 3 / 109 (2.8) | 4 / 120 (3.3) | 0.60 (0.12 – 3.08) | 0.54 |
|  |  | | | contemplation | | |  | 27 / 109 (24.8) | 34 / 120 (28.3) | 0.64 (0.128 – 1.46) | 0.28 |
|  |  | | | preparation | | |  | 38 / 109 (34.9) | 36 / 120 (30.0) | 0.84 (0.38 – 1.88) | 0.68 |
|  |  | | | Action | | |  | 21 / 109 (19.3) | 30 / 120 (25.0) | 0.56 (0.24 – 1.33) | 0.19 |
|  |  | | | maintenance | | |  | 20 / 109 (18.3) | 16 / 120 (13.3) | *[ref]* | *[ref]* |
|  | Previous support in weight loss | | | | | |  |  |  |  |  |
|  |  | | | | by dietician | | 207 | 40 / 98 (40.8) | 60 / 109 (55.0) | 0.56 (0.34 – 0.98) | **0.04** |
|  |  | | | | by partner | | 207 | 56 / 98 (57.1) | 63 / 109 (5.8) | 0.97 (0.56 – 1.69) | 0.92 |
|  |  | | | | no social support | | 207 | 38 / 98 (38.8) | 34 / 109 (31.2) | 1.40 (0.79 – 2.48) | 0.25 |
|  | Self-efficacy | | | | | | 227 |  |  |  | 0.14 |
|  |  | | | | extremely unlikely | |  | 1 / 108 (0.9) | 0 / 119 (0.0) | *n.a.* | *n.a.* |
|  |  | | | | Unlikely | |  | 2 / 108 (1.9) | 4 / 119 (3.4) | 0.41 (0.07 – 2.51) | 0.34 |
|  |  | | | | do not know / neutral | |  | 35 / 108 (32.4) | 58 / 119 (48.7) | 0.50 (0.24 – 1.04) | 0.07 |
|  |  | | | | Likely | |  | 47 / 108 (43.5) | 38 / 119 (31.9) | 1.02 (0.49 – 2.15) | 0.96 |
|  |  | | | | extremely likely | |  | 23 / 108 (21.3) | 19 / 119 (16.0) | [ref] | [ref] |
|  | Body satisfaction | | | | | | 232 |  |  |  | 0.91 |
|  |  | | | | no, never | |  | 48 / 111 (43.2) | 59 / 121 (48.8) | [ref] | [ref] |
|  |  | | | | no, mostly not | |  | 44 / 111 (39.6) | 42 / 121 (34.7) | 1.29 (0.72 – 2.28) | 0.38 |
|  |  | | | | sometimes, sometimes not | |  | 15 / 111 (13.5) | 16 / 121 (13.2) | 1.15 (0.52 – 2.57) | 0.73 |
|  |  | | | | yes, mostly | |  | 4 / 111 (3.6) | 3 / 121 (2.5) | 1.64 (0.35 – 7.68) | 0.53 |
|  |  | | | | yes, always | |  | 0 / 111 (0.0) | 1 / 121 (0.8) | *n.a.* | *n.a.* |
|  | Dutch Eating Behavior Questionnaire | | | | | |  |  |  |  |  |
|  |  | emotional eating (13-65 points) | | | | | 250 | 36.0 (28.3 – 44.0) | 36.0 (25.8 – 44.0) | 1.00 (0.98– 1.02) | 0.86 |
|  |  | external eating (10-50 points) | | | | | 250 | 30.0 (25.0 – 33.0) | 27.0 (22.0 – 32.0) | 1.07 (1.02 – 1.12) | **<0.01** |
|  |  | restrained eating (10-50 points) | | | | | 252 | 31.0 (28.0 – 365.0) | 31.0 (28.0 – 35.0) | 1.00 (0.96 – 1.05) | 0.89 |
|  |  | | | | | |  |  |  |  |  |
| *Diet and physical activity characteristics* | Mean energy intake (kcal/day) | | | | | | 210 | 1920 (1590 – 2200) | 1883 (1600 – 2100) | 0.99 (0.94 – 1.065) ^b^ | 0.81 |
|  | Mean steps (day) | | | | | | 226 | 6000 (4000 – 8000) | 6000 (4000 – 8000) | 1.00 (0.893 – 1.09) ^c^ | 085 |
|  | Frequency of breakfast use (days / week) | | | | | | 237 | 7 (4 – 7) | 7 (5 – 7) | 0.94 (0.84 – 1.05) | 0.28 |
|  | Vegetable intake (grams/day) | | | | | | 237 | 114 (79– 164) | 114 (86 – 157) | 1.00 (0.96 – 1.04) ^d^ | 0.96 |
|  | Fruit intake (grams/day) | | | | | | 237 | 100 (43 – 171) | 85 (57 – 171) | 0.99 (0.97 – 1.02) ^d^ | 0.66 |
|  | Meeting recommendations vegetable intake ^5^ | | | | | | 237 | 20 / 113 (17.7) | 22 / 124 (17.7) | 1.00 (0.51 – 1.94) | 0.99 |
|  | Meeting recommendations fruit intake ^5^ | | | | | | 237 | 23 / 113 (20.4) | 27 / 124 (21.8) | 0.92 (0.49 – 1.72) | 0.79 |
|  | Meeting recommendations fruit and juice intake ^6^ | | | | | | 233 | 33 / 111 (29.7) | 34 / 122 (27.9) | 1.10 (0.62 – 1.93) | 0.75 |
|  | Meeting recommendations fruit- and vegetable intake ^5^ | | | | | | 231 | 8 / 110 (7.3) | 11 / 121 (9.1) | 0.78 (0.30 – 2.03) ^e^ | 0.61 |
|  | Alcoholic beverages (units / day) | | | | | | 218 | 0.00 (0.00 – 0.18) | 0.00 (0.00 – 0.18) | 1.40 (0.72 – 2.69) | 0.32 |
|  | Total moderate to vigorous physical activity (min/week) | | | | | | 234 | 300 (120 – 784) | 382 (150 – 855) | 1.00 (0.99 – 1.01) | 0.47 |
|  | Meeting recommendations guideline physical activity | | | | | | 232 | 75 / 110 (68.2) | 95 / 122 (77.9) | 0.61 (0.34 – 1.10) | 0.10 |
| *Partner characteristics* | Age (years) | | | | | | 261 | 33.6 (29.6 – 36.7) | 33.4 (29.9 – 37.0) | 1.01 (0.97 – 1.05) | 0.67 |
|  | BMI (kg/m^2^) | | | | | | 226 | 27.8 (24.5 – 31.3) | 27.8 (24.7 – 31.1) | 1.00 (0.96 – 1.05) | 0.97 |
|  | Non-Caucasian | | | | | | 261 | 11 / 119 (9.2) | 12 / 142 (8.5) | 1.10 (0.427 – 2.60) | 0.82 |
|  | Smoking | | | | | | 260 | 40 / 119 (33.6) | 61 / 141 (43.3) | 0.66 (0.40 – 1.10) | 0.11 |
| All determinants were assessed at baseline. Results of univariable logistic regression analyses on determinants of successful weight loss. Successful weight loss is defined as loss of ≥5% of original body weight or reaching a BMI <29kg/m^2^. ORs indicate the odds of successful weight loss given the determinant is present. ^a^ Waist-hip ratio: per 0.10 increase. ^b^ Baseline calorie intake: per 100 kcal increase. ^c^ Baseline step counts: per 1000 steps increase. ^d^ Baseline vegetable and fruit intake: per 10 grams / day increase. ^e^ Total moderate to vigorous physical activity: per 30 min / week increase. ^1^ Socioeconomic status score (Netherlands Institute for Social Research (SCP)) in year 2010 relative to Dutch average of 0, a higher score represents a higher socioeconomic status. ^2^ Weight change during the first 1,5 months of the intervention program. ^3^ Calculated upon the highest and lowest weight in the past 10 years. ^4^ Measured by the Short-Form-36 Questionnaire. ^5^ Dutch Guidelines for a healthy diet 2006: minimal recommended fruit and vegetable intake 200 grams per day. ^6^ Dutch Guidelines for a healthy diet 2006: of minimal recommended fruit intake of 200 grams/day a maximum of 100 grams of fruit can be substituted by 1 glass of fruit juice daily. N.a.: not applicable. | | | | | | | | | | | |

# Additional Table S2. Determinants of continuous weight change, calorie intake and step counts

| **Category** | **Determinant** | | | | | | **Weight loss** | | | | **Energy intake** | | **Number of steps** | | |
| --- | --- | --- | --- | --- | --- | --- | --- | --- | --- | --- | --- | --- | --- | --- | --- |
|  |  | | | | | | **β kg**  **(95% CI)** | | | ***p*** | **β (95% CI) x 100 kcal** | ***p*** | **Β (95% CI) x 1000 steps** | | ***p*** |
| *Demographic characteristics* | Age of women (years) | | | | | | 0.04 (-0.04 – 0.12) | | | 0.35 | 0.06 (-0.00 – 0.12) | 0.06 | 0.01 (-0.09 – 0.07) | | 0.81 |
|  | Non-Caucasian | | | | | | 0.63 (-0.61 – 1.87) | | | 0.32 | 0.34 (-0.64 – 1.33) | 0.49 | -0.79 (-1.94 – 0.36) | | 0.18 |
|  | Education level | | | | | |  | | | 0.11 |  | 0.91 |  | | 0.22 |
|  |  | | | | | no education / primary school | *[ref]* | | | *[ref]* | *[ref]* | *[ref]* | *[ref]* | | *[ref]* |
|  |  | | | | | secondary education | -0.52 (-2.37 – 1.33) | | | 0.58 | -0.59 (-2.31 – 1.12) | 0.50 | 1.05 (-0.98 – 3.08) | | 0.31 |
|  |  | | | | | intermediate vocational education | -1.19 (-2.95 – 0.57) | | | 0.19 | -0.42 (-2.08 – 1.24) | 0.62 | 0.94 (-1.04 – 2.91) | | 0.35 |
|  |  | | | | | higher vocational education and university | -1.69 (-3.56 – 0.18) | | | 0.08 | -0.48 (-2.19 – 1.23) | 0.58 | 1.76 (-0.30 – 3.82) | | 0.09 |
|  | Socioeconomic status score ^1^ | | | | | | -0.32 (-0.70 – 0.06) | | | 0.10 | 0.10 (-0.15 – 0.34) | 0.45 | -0.18 (-0.52 – 0.16) | | 0.29 |
|  | Current smoker | | | | | | -0.12 (1.00 – 0.76) | | | 0.79 | -0.34 (-0.97 – 0.30) | 0.30 | -0.25 (-1.07 – 0.58) | | 0.56 |
|  |  | | | | | |  | | |  |  |  |  | |  |
| *Infertility characteristics* | Duration of infertility (months) | | | | | | 0.02 (0.00 – 0.03) | | | **0.03** | 0.01 (-0.00 – 0.03) | 0.06 | -0.01 (-0.03 – 0.01) | | 0.24 |
|  | Nulliparous | | | | | | -0.17 (-1.21 – 0.87) | | | 0.75 | -0.10 (-0.82 – 0.61) | 0.78 | 0.01 (-0.98 – 1.00) | | 0.99 |
|  | Anovulatory | | | | | | 0.43 (-0.34 – 1.19) | | | 0.27 | -0.24 (-0.79 – 0.30) | 0.38 | 0.07 (-0.65 – 0.80) | | 0.84 |
|  | Male factor infertility | | | | | | 0.18 (-0.72 – 1.07) | | | 0.70 | 0.02 (-0.58 – 0.62) | 0.95 | 0.06 (-0.77 – 0.89) | | 0.89 |
|  | Unexplained infertility | | | | | | -0.33 (-1.16 – 0.49) | | | 0.43 | 0.09 (-0.50 – 0.67) | 0.77 | -0.22 (-0.99 – 0.56) | | 0.59 |
|  |  | | | | | |  | | |  |  |  |  | |  |
| *Anthropometric and weight characteristics* | BMI (kg/m^2^) | | | | | | 0.25 (0.08 – 0.42) | | | **<0.01** | 0.04 (-0.04 – 0.12) | 0.29 | -0.05 (-0.15 – 0.06) | | 0.38 |
|  | Waist circumference (cm) | | | | | | 0.04 (-0.01 – 0.09) | | | 0.10 | -0.00 (-0.03 – 0.03) | 0.95 | -0.01 (-0.05 – 0.03) | | 0.55 |
|  | Waist / hip ratio | | | | | | -0.10 (-0.69 – 0.49) ^a^ | | | 0.74 | -0.28 (-0.70 – 0.13) ^a^ | 0.18 | 0.06 (-0.51 – 0.63) ^a^ | | 0.84 |
|  | Highest weight in past 10 years (kg) | | | | | | 0.04 (-0.02 – 0.11) | | | 0.21 | 0.01 (-0.01 – 0.03) | 0.41 | -0.01 (-0.03 – 0.01) | | 0.47 |
|  | Highest weight ever | | | | | |  | | | 0.13 |  | 0.98 |  | | 0.24 |
|  |  | same as current weight | | | | | *[ref]* | | | *[ref]* | *[ref]* | *[ref]* | *[ref]* | | *[ref]* |
|  |  | 1 – 5 kg above current weight | | | | | -0.60 (-1.74 – 0.53) | | | 0.30 | -0.01 (-0.74 – 0.77 | 0.98 | -0.93 (-1.90 – 0.05) | | 0.06 |
|  |  | 6 – 10kg above current weight | | | | | -0.16 (-1.50 – 1.18) | | | 0.81 | -0.01 (-0.88 – 0.86) | 0.98 | -0.21 (-1.33 – 0.92) | | 0.72 |
|  |  | > 10 kg above current weight | | | | | 0.87 (-0.43 – 2.18) | | | 0.19 | -0.16 (-1.02 – 0.70) | 0.72 | -0.69 (-1.78 – 0.39) | | 0.21 |
|  | Weight variability in past 10 years ^3^ (kg) | | | | | | -0.00 (-0.04 – 0.03) | | | 0.80 | -0.02 (-0.04 – 0.00) | 0.09 | 0.02 (-0.00 – 0.05) | | 0.07 |
|  | Number of weight loss attempts in past 5 years | | | | | |  | | | **<0.01** |  | 0.81 |  | | 0.62 |
|  |  | | none | | | | *[ref]* | | | *[ref]* | *[ref]* | *[ref]* | | *[ref]* | *[ref]* |
|  |  | | 1 attempt | | | | 0.80 (-1.09 – 2.69) | | | 0.40 | 0.17 (-1.08 – 1.43) | 0.79 | | 0.74 (-0.87 – 2.36) | 0.37 |
|  |  | | 2 – 3 attempts | | | | 0.47 (-1.28 – 2.22) | | | 0.60 | 0.35 (-0.81 – 1.50) | 0.56 | | -0.13 (-1.63 – 1.37) | 0.86 |
|  |  | | 4 – 5 attempts | | | | 0.76 (-1.10 – 2.62) | | | 0.42 | 0.51 (-0.71 – 1.74) | 0.41 | | 0.07 (-1.52 – 1.65) | 0.93 |
|  |  | | > 5 attempts | | | | 2.26 (0.57 – 3.95) | | | **<0.01** | 0.56 (-0.55 – 1.68) | 0.32 | | -0.10 (-1.56 – 1.37) | 0.89 |
|  |  | | | | | |  | | |  |  |  | |  |  |
| *Metabolic characteristics* | Insulin resistance (HOMA-IR) | | | | | | 0.15 (-0.06 – 0.36) | | | 0.15 | -0.05 (-0.19 – 0.08) | 0.44 | | 0.19 (0.02 – 0.37) | **0.03** |
|  | Metabolic syndrome | | | | | | -0.21 (-1.07 – 0.65) | | | 0.63 | -0.17 (-0.74 – 0.39) | 0.54 | | 1.05 (0.32 – 1.78) | **<0.01** |
|  |  | | | | | |  | | |  |  |  | |  |  |
| *Psychosocial characteristics* | Quality of Life ^4^ | | | | | | |  | |  |  |  | |  |  |
|  |  | | | physical component (score) | | | | -0.04 (-0.09 – 0.00) | | 0.08 | 0.00 (-0.03 – 0.03) | 0.80 | | 0.02 (-0.01 – 0.06) | 0.21 |
|  |  | | | mental component (score) | | | | -0.04 (-0.09 – 0.01) | | 0.08 | -0.01 (-0.05 – 0.02) | 0.41 | | -0.02 (-0.06 – 0.03) | 0.44 |
|  | Readiness to change: weight loss | | | | | | |  | | **0.001** |  | 0.81 | |  | 0.51 |
|  |  | | | precontemplation | | | | *n.a.* | | *n.a.* | *n.a.* | *n.a.* | | *n.a.* | *n.a.* |
|  |  | | | contemplation | | | | -2.21 (-6.73 – 2.32) | | 0.34 | -0.25 (-4.12 – 3.62) | 0.90 | | 0.59 (-2.97 – 4.14) | 0.75 |
|  |  | | | preparation | | | | -1.11 (-2.35 – 0.13) | | 0.08 | -0.42 (-1.28 – 0.43) | 0.33 | | 0.79 (-0.28 – 1.86) | 0.15 |
|  |  | | | action | | | | -1.90 (-2.81 – -0.99) | | **<0.001** | -0.14 (-0.77 – 0.49) | 0.66 | | 0.41 (-0.40 – 1.21) | 0.32 |
|  |  | | | maintenance | | | | *[ref]* | | *[ref]* | *[ref]* | *[ref]* | | *[ref]* | *[ref]* |
|  | Readiness to change: physical activity | | | | | | |  | | 0.40 |  | 0.87 | |  | **0.04** |
|  |  | | | precontemplation | | | | 1.31 (-0.96 – 3.58) | | 0.26 | -0.73 (-2.54 – 1.07) | 0.42 | | -0.18 (-2.37 – 2.00) | 0.87 |
|  |  | | | contemplation | | | | 1.29 (-0.01 – 2.60) | | 0.053 | -0.43 (-1.33 – 0.46) | 0.34 | | -0.35 (-1.45– 0.76) | 0.53 |
|  |  | | | preparation | | | | 0.85 (-0.40 – 2.10) | | 0.18 | -0.33 (-1.18 – 0.52) | 0.44 | | 0.03 (-1.04 – 1.11) | 0.95 |
|  |  | | | action | | | | 0.74 (-0.61 – 2.08) | | 0.28 | -0.28 (-1.19 – 0.63) | 0.54 | | 1.23 (0.09 – 2.37) | **0.04** |
|  |  | | | maintenance | | | | *[ref]* | | *[ref]* | *[ref]* | *[ref]* | | *[ref]* | *[ref]* |
|  | Previous support in weight loss | | | | | | |  | |  |  |  | |  |  |
|  |  | | | | by dietician | | | 1.37 (0.48 – 2.27) | | **<0.01** | 0.47 (-0.13 – 1.07) | 0.12 | | 0.18 (-0.58 – 0.94) | 0.64 |
|  |  | | | | by partner | | | 0.08 (-0.83 – 1.00) | | 0.86 | 0.30 (-0.31 – 0.91) | 0.33 | | 0.34 (-1.11 – 0.42) | 0.37 |
|  |  | | | | no social support | | | 0.09 (-0.87 – 1.05) | | 0.85 | -0.54 (-1.16 – 0.08) | 0.09 | | 0.13 (-0.67– 0.91) | 0.75 |
|  | Self-efficacy | | | | | | |  | | 0.06 |  | **<0.01** | |  | 0.52 |
|  |  | | | | extremely unlikely | | | *[ref]* | | *[ref]* | *[ref]* | *[ref]* | | *[ref]* | *[ref]* |
|  |  | | | | unlikely | | | 2.64 (-4.31 – 9.59) | | 0.46 | -3.31 (-7.48 – 0.87) | 0.12 | | 0.53 (-4.97 – 6.03) | 0.85 |
|  |  | | | | do not know / neutral | | | 1.99 (-4.49 – 8.48) | | 0.55 | -2.75 (-6.65 – 1.15) | 0.17 | | 1.24 (-3.81 – 6.29) | 0.63 |
|  |  | | | | likely | | | 0.92 (-5.57 – 7.41) | | 0.78 | -2.41 (-6.31 – 1.50) | 0.23 | | 1.65 (-3.40 – 6.70) | 0.52 |
|  |  | | | | extremely likely | | | 0.49 (-6.04 – 7.02) | | 0.88 | -3.75 (-7.69 – 0.19) | 0.06 | | 0.88 (-4.21 – 5.96) | 0.73 |
|  | Body satisfaction | | | | | | |  | | 0.39 |  | 0.47 | |  | 0.65 |
|  |  | | | | no, never | | | *[ref]* | | *[ref]* | *[ref]* | *[ref]* | | *[ref]* | *[ref]* |
|  |  | | | | no, mostly not | | | -0.23 (-1.17 – 0.71) | | 0.63 | 0.41 (-0.21 – 1.03) | 0.19 | | -0.43 (-1.22 – 0.37) | 0.29 |
|  |  | | | | sometimes, sometimes not | | | -0.21 (-1.52 – 1.10) | | 0.75 | 0.29 (-0.61 – 1.20) | 0.52 | | 0.12 (-0.97 – 1.21) | 0.83 |
|  |  | | | | yes, mostly | | | -0.58 (-3.17 – 2.00) | | 0.66 | 1.14 (-0.45 – 2.72) | 0.16 | | 0.77 (-1.46 – 2.98) | 0.50 |
|  |  | | | | yes, always | | | 6.19 (-0.24 – 12.62) | | 0.06 | 1.41 (-2.24 – 5.05) | 0.45 | | -1.42 (-6.21 – 3.38) | 0.56 |
|  | Dutch Eating Behavior Questionnaire | | | | | | |  | | | | | | | |
|  |  | emotional eating (13-65 points) | | | | | | -0.01 (-0.04 – 0.03) | 0.71 | | 0.02 (0.00 – 0.04) | 0.11 | | -0.03 (-0.06 – 0.01) | 0.11 |
|  |  | external eating (10-50 points) | | | | | | -0.01 (-0.08 – 0.05) | 0.67 | | 0.02 (-0.03 – 0.06) | 0.53 | | -0.04 (-0.10 – 0.02) | 0.24 |
|  |  | restrained eating (10-50 points) | | | | | | 0.03 (-0.04 – 0.09) | 0.43 | | -0.00 (-0.05 – 0.05) | 0.99 | | 0.05 (-0.01 – 0.12) | 0.09 |
|  |  | | | | | | | | | | | | | | |
| *Diet and physical activity characteristics* | Mean energy intake (kcal/day) | | | | | | | 0.05 (-0.05 – 0.14) ^b^ | 0.32 | | n.a. | n.a. | | 0.06 (-0.02 – 0.14) ^b^ | 0.13 |
|  | Mean steps (day) | | | | | | | -0.06 (-0.18 – 0.05) ^c^ | 0.27 | | -0.06 (-0.15 – 0.02) ^c^ | 0.16 | | n.a. | n.a. |
|  | Frequency of breakfast use (days / week) | | | | | | | -0.09 (-0.27 – 0.09) | 0.34 | | 0.07 (-0.06 – 0.20) | 0.29 | | 0.06 (-0.10 – 0.21) | 0.47 |
|  | Vegetable intake (grams/day) | | | | | | | -0.01 (-0.08 – 0.05) ^d^ | 0.64 | | 0.00 (-0.04 – 0.04) ^d^ | 0.89 | | 0.03 (-0.02 – 0.08) ^d^ | 0.27 |
|  | Fruit intake (grams/day) | | | | | | | -0.01 (-0.05 – 0.04) ^d^ | 0.79 | | 0.02 (-0.02 – 0.05) ^d^ | 0.38 | | 0.03 (0.01 – 0.07) ^d^ | 0.10 |
|  | Meeting recommendations vegetable intake ^5^ | | | | | | | 0.28 (-0.79 – 1.35) | 0.61 | | -0.07 (-0.80 – 0.67) | 0.86 | | 0.78 (-0.11 – 1.67) | 0.09 |
|  | Meeting recommendations fruit intake ^5^ | | | | | | | -0.20 (-1.20 – 0.80) | 0.69 | | 0.45 (-0.24 – 1.15) | 0.20 | | 0.16 (-0.70 – 1.02) | 0.72 |
|  | Meeting recommendations fruit and juice intake ^6^ | | | | | | | -0.32 (-1.23 – 0.59) | 0.49 | | 0.49 (-0.14 – 1.12) | 0.12 | | 0.24 (-0.53 – 1.01) | 0.54 |
|  | Meeting recommendations fruit- and vegetable intake ^5^ | | | | | | | 0.98 (-0.52 – 2.47) | 0.20 | | 0.87 (-0.15 – 1.89) | 0.10 | | 0.93 (-0.36 – 2.22) | 0.16 |
|  | Alcoholic beverages (units / day) | | | | | | | 0.42 (-0.61 – 1.45) | 0.42 | | -0.57 (-1.22 – 0.09) | 0.09 | | 0.13 (-0.69 – 0.95) | 0.75 |
|  | Total moderate to vigorous physical activity (min/week) | | | | | | | -0.00 (-0.02 – 0.02) ^e^ | 0.80 | | -0.01 (-0.02 – 0.00) ^e^ | 0.10 | | 0.01 (-0.01 – 0.03) ^e^ | 0.18 |
|  | Meeting recommendations guideline physical activity | | | | | | | -0.26 (-1.17 – 0.64) | 0.57 | | -0.49 (-1.09 – 0.11) | 0.11 | | 0.25 (-0.53 – 1.04) | 0.52 |
|  |  | | | | | | |  |  | |  |  | |  |  |
| *Partner characteristics* | Age (years) | | | | | | | -0.00 (-0.06 – 0.06) | 0.99 | | 0.06 (0.01 – 0.11) | **0.01** | | 0.01 (-0.06 – 0.05) | 0.86 |
|  | Baseline BMI (kg/m^2^) | | | | | | | -0.03 (-0.11 – 0.04) | 0.38 | | 0.01 (-0.03 – 0.06) | 0.60 | | -0.04 (-0.12 – 0.03) | 0.25 |
|  | Non-Caucasian | | | | | | | -0.38 (-1.74 – 0.99) | 0.59 | | 0.83 (-0.34 – 1.99) | 0.16 | | -0.50 (-1.85 – 0.86) | 0.47 |
|  | Smoking | | | | | | | 0.40 (-0.38 – 1.18) | 0.31 | | -0.16 (-0.72 – 0.41) | 0.59 | | -0.12 (-0.85 – 0.60) | 0.74 |
|  |  | | | | | | | | | | | | | | |
| All determinants were assessed at baseline. Results of univariable mixed effects regressions models on determinants of weight loss, energy intake and the number of steps. With correction for baseline BMI or for baseline energy intake or steps and including a random intercept. A negative number indicates additional weight loss or a decrease in the intake of kcal/steps. A positive number indicates less weight loss or an increase the intake of kcal/steps. β: regression coefficient. ^a^ Waist-hip ratio: per 0.10 increase. ^b^ Baseline calorie intake: per 100 kcal increase. ^c^ Baseline step counts: per 1000 steps increase. ^d^ Baseline vegetable and fruit intake: per 10 grams / day increase. ^e^ Total moderate to vigorous physical activity: per 30 min / week increase. ^1^ Socioeconomic status score (Netherlands Institute for Social Research (SCP)) in year 2010 relative to Dutch average of 0, a higher score represents a higher socioeconomic status. ^2^ Weight change during the first 1,5 months of the intervention program. ^3^ Calculated upon the highest and lowest weight in the past 10 years. ^4^ Measured by the Short-Form-36 Questionnaire. ^5^ Dutch Guidelines for a healthy diet 2006: minimal recommended fruit and vegetable intake 200 grams per day. ^6^ Dutch Guidelines for a healthy diet 2006: of minimal recommended fruit intake of 200 grams/day a maximum of 100 grams of fruit can be substituted by 1 glass of fruit juice daily. N.a.: not applicable. | | | | | | | | | | | | | | | |

# Additional Table S3. Determinants of completion of the lifestyle intervention

| **Category** | **Determinant** | | | | | | **N** | **Completer**  **(median, IQR or N, %)** | **Non-completer**  **(median, IQR or N, %)** | **Odds ratio (95% CI)** | ***p*** |  |
| --- | --- | --- | --- | --- | --- | --- | --- | --- | --- | --- | --- | --- |
| *Demographic characteristics* | Age of women (years) | | | | | | 289 | 29.7 (26.6 – 33.5) | 29.4 (24.9 – 32.0) | 1.05 (0.98 – 1.12) | 0.15 |  |
|  | Non-Caucasian | | | | | | 289 | 23 / 226 (10.2) | 10 / 63 (15.9) | 0.60 (0.27 – 1.34) | 0.21 |  |
|  | Education level | | | | | | 276 |  |  |  | 0.08 |  |
|  |  | | | | | no education / primary school |  | 13 / 214 (6.1) | 4 / 62 (6.5) | [ref] | [ref] |  |
|  |  | | | | | secondary education |  | 46 / 214 (21.5) | 20 / 62 (32.3) | 0.71 (0.21 – 2.44) | 0.58 |  |
|  |  | | | | | intermediate vocational education |  | 103 / 214 (48.1) | 32 / 62 (51.6) | 0.99 (0.30 – 3.25) | 0.99 |  |
|  |  | | | | | higher vocational education and university |  | 52 / 214 (24.3) | 6 / 62 (9.7) | 2.67 (0.66 – 10.9) | 0.17 |  |
|  | Socioeconomic status score ^1^ | | | | | | 230 | -0.53 (-1.20 – 0.39) | -0.43 (-1.48 – 0.36) | 1.01 (0.77 – 1.34) | 0.93 |  |
|  | Current smoker | | | | | | 285 | 53 / 222 (23.9) | 23 / 63 (36.5) | 0.55 (0.30 – 0.99) | **0.047** |  |
|  |  | | | | | |  |  |  |  |  |  |
| *Infertility characteristics* | Duration of infertility (months) | | | | | | 289 | 23.0 (15.0 – 36.3) | 18.0 (14.0 – 36.0) | 1.01 (0.99 – 1.02) | 0.40 |  |
|  | Nulliparous | | | | | | 276 | 180 / 213 (84.5) | 46 / 63 (73.0) | 2.02 (1.03 – 3.93) | **0.04** |  |
|  | Anovulatory | | | | | | 288 | 100 / 225 (44.4) | 28 / 63 (44.4) | 1.00 (0.57 – 1.76) | 1.00 |  |
|  | Male factor infertility | | | | | | 288 | 56 / 225 (24.9) | 11 / 63 (17.5) | 1.57 (0.77 – 3.21) | 0.22 |  |
|  | Unexplained infertility | | | | | | 288 | 68 / 225 (30.3) | 18 / 63 (28.6) | 1.08 (0.59 – 2.01) | 0.80 |  |
|  |  | | | | | |  |  |  |  |  |  |
| *Anthropometric and weight characteristics* | BMI (kg/m^2^) | | | | | | 288 | 36.3 (33.5 – 38.3) | 35.5 (33.3 – 38.8) | 1.04 (0.95 – 1.13) | 0.41 |  |
|  | Waist circumference (cm) | | | | | | 262 | 107.0 (102.0 – 113.0) | 107.0 (99.0 – 115.0) | 1.01 (0.98 – 1.05) | 0.42 |  |
|  | Waist/hip ratio | | | | | | 280 | 0.86 (0.82 – 0.91) | 0.88 (0.82 – 0.93) | 0.71 (0.47 – 1.09) | 0.12 |  |
|  | Highest weight in past 10 years (kg) | | | | | | 246 | 108.0 (98.0 – 119.0) | 105.0 (95.0 – 119.0) | 1.00 (0.99 – 1.03) | 0.64 |  |
|  | Highest weight ever | | | | | | 235 |  |  |  | 0.36 |  |
|  |  | same as current weight | | | | |  | 45 / 191 (23.6) | 14 / 44 (31.8) | *[ref]* | *[ref]* |  |
|  |  | 1 – 5 kg above current weight | | | | |  | 67 / 191 (35.1) | 18 / 44 (40.9) | 1.16 (0.52 – 2.56) | 0.72 |  |
|  |  | 6 – 10kg above current weight | | | | |  | 39 / 191 (20.4) | 5 / 44 (11.4) | 2.43 (0.80 – 7.35) | 0.12 |  |
|  |  | > 10 kg above current weight | | | | |  | 40 / 191 (20.9) | 7 / 44 (15.9) | 1.78 (0.65 – 4.84) | 0.26 |  |
|  | Weight variability in past 10 years ^3^ (kg) | | | | | | 241 | 30.0 (21.0 – 39.0) | 30.0 (21.0 – 41.5) | 1.00 (0.97 – 1.02) | 0.80 |  |
|  | Number of weight loss attempts in past 5 years | | | | | | 250 |  |  |  | 0.58 |  |
|  |  | | none | | | |  | 13 / 201 (6.5) | 4 / 49 (8.2) | *[ref]* | *[ref]* |  |
|  |  | | 1 attempt | | | |  | 29 / 201 (14.4) | 6 / 49 (12.2) | 1.49 (0.36 – 6.18) | 0.59 |  |
|  |  | | 2 – 3 attempts | | | |  | 57 / 201 (28.4) | 9 / 49 (18.4) | 1.95 (0.52 – 7.32) | 0.32 |  |
|  |  | | 4 – 5 attempts | | | |  | 29 / 201 (14.4) | 10 / 49 (20.4) | 0.89 (0.24 – 3.38) | 0.87 |  |
|  |  | | > 5 attempts | | | |  | 73 / 201 (36.3) | 20 / 49 (40.8) | 1.12 (0.33 – 3.82) | 0.85 |  |
|  |  |  | | | | |  |  |  |  |  |  |
| *Metabolic characteristics* | Insulin resistance (HOMA-IR) | | | | | | 247 | 2.90 (1.9 – 3.9) | 2.8 (2.4 – 3.3) | 0.91 (0.79 – 1.05) | 0.19 |  |
|  | Metabolic syndrome | | | | | | 237 | 94 / 187 (50.3) | 29 / 50 (58.0) | 0.73 (0.39 – 1.38) | 0.33 |  |
|  |  | | | | | |  |  |  |  |  |  |
| *Psychosocial characteristics* | Quality of Life ^4^ | | | | | |  |  |  |  |  |  |
|  |  | | | physical component (score) | | | 236 | - 1. (46.3 – 55.5) | 52.2 (46.9 – 54.7) | 0.99 (0.96 – 1.03) | 0.70 |  |
|  |  | | | mental component (score) | | | 236 | 52.7 (46.7 – 56.0) | 52.8 (42.4 – 56.3) | 1.02 (0.98 – 1.05) | 0.34 |  |
|  | Readiness to change: weight loss | | | | | | 246 |  |  |  | **0.01** |  |
|  |  | | | precontemplation | | |  | 0 / 198 (0.0) | 0 / 48 (0.0) | *n.a.* | n.a. |  |
|  |  | | | contemplation | | |  | 0 / 198 (0.0) | 2 / 48 (4.2) | *n.a.* | n.a. |  |
|  |  | | | preparation | | |  | 24 / 198 (12.1) | 14 / 48 (29.2) | 0.43 (0.19 – 1.00) | **0.049** |  |
|  |  | | | action | | |  | 99 / 198 (50.0) | 13 / 48 (27.1) | 1.93 (0.90 – 4.15) | 0.09 |  |
|  |  | | | maintenance | | |  | 75 / 198 (37.9) | 19 / 48 (39.6) | *[ref]* | *[ref]* |  |
|  | Readiness to change: physical activity | | | | | | 249 |  |  |  | 0.08 |  |
|  |  | | | precontemplation | | |  | 5 / 198 (2.5) | 6 / 51 (11.8) | 0.12 (0.03 – 0.56) | <0.01 |  |
|  |  | | | contemplation | | |  | 52 / 198 (26.3) | 14 / 51 (27.5) | 0.55 (0.18 – 1.66) | 0.29 |  |
|  |  | | | preparation | | |  | 65 / 198 (32.8) | 14 / 51 (27.5) | 0.68 (0.23 – 2.06) | 0.50 |  |
|  |  | | | action | | |  | 42 / 198 (21.2) | 12 / 51 (23.5) | 0.52 (0.17 – 1.61) | 0.25 |  |
|  |  | | | maintenance | | |  | 34 / 198 (17.2) | 5 / 51 (9.8) | *[ref]* | *[ref]* |  |
|  | Previous support in weight loss | | | | | |  |  |  |  |  |  |
|  |  | | | | by dietician | | 224 | 87 / 182 (47.8) | 20 / 42 (47.6) | 1.01 (0.52 – 1.97) | 0.98 |  |
|  |  | | | | by partner | | 224 | 105 / 182 (57.7) | 26 / 42 (61.9) | 0.84 (0.42 – 1.67) | 0.62 |  |
|  |  | | | | no social support | | 224 | 65 / 182 (35.7) | 11 / 42 (26.2) | 1.57 (0.74 – 3.32) | 0.24 |  |
|  | Self-efficacy | | | | | | 245 |  |  |  | 0.97 |  |
|  |  | | | | extremely unlikely | |  | 0 / 197 (0.0) | 1 / 48 (2.1) | *n.a.* | *n.a.* |  |
|  |  | | | | unlikely | |  | 6 / 197 (3.0) | 0 / 48 (0.0) | *n.a.* | *n.a.* |  |
|  |  | | | | do not know / neutral | |  | 83 / 197 (42.1) | 22 / 48 (45.8) | 0.71 (0.28 – 1.82) | 0.48 |  |
|  |  | | | | likely | |  | 71 / 197 (36.0) | 18 / 48 (37.5) | 0.75 (0.29 – 1.95) | 0.55 |  |
|  |  | | | | extremely likely | |  | 37 / 197 (18.8) | 7 / 48 (14.6) | *[ref]* | *[ref]* |  |
|  | Body satisfaction | | | | | | 250 |  |  |  | 0.88 |  |
|  |  | | | | no, never | |  | 91 / 201 (45.3) | 25 / 49 (51.0) | *[ref]* | *[ref]* |  |
|  |  | | | | no, mostly not | |  | 76 / 201 (37.8) | 15 / 49 (30.6) | *n.a.* | *n.a.* |  |
|  |  | | | | sometimes, sometimes not | |  | 28 / 201 (13.9) | 7 / 49 (14.3) | 0.69 (0.13 – 3.75) | 0.67 |  |
|  |  | | | | yes, mostly | |  | 5 / 201 (2.5) | 2 / 49 (4.1) | 1.10 (0.343 – 2.81) | 0.84 |  |
|  |  | | | | yes, always | |  | 1 / 201 (0.4) | 0 / 49 (0.0) | 1.39 (0.69 – 2.83) | 0.36 |  |
|  | Dutch Eating Behavior Questionnaire | | | | | |  |  |  |  |  |  |
|  |  | emotional eating (13-65 points) | | | | | 276 | 36.0 (27.0 – 44.0) | 37.0 (25.8 – 45.0) | 1.00 (0.98 – 1.03) | 0.96 |  |
|  |  | external eating (10-50 points) | | | | | 277 | 28.0 (24.0 – 32.0) | 29.0 (25.0 – 33.0) | 0.98 (0.93 – 1.03) | 0.48 |  |
|  |  | restrained eating (10-50 points) | | | | | 278 | 32.0 (28.0 – 36.0) | 31.0 (26.0 – 33.0) | 1.07 (1.02 – 1.12) | **0.01** |  |
|  |  | | | | | |  |  |  |  |  |  |
| *Diet and physical activity characteristics* | Mean energy intake (kcal/day) | | | | | | 213 | 1868 (1568 – 2200) | 2000 (1687 – 2300) | 0.96 (0.89 – 1.03) | 0.28 |  |
|  | Mean steps (day) | | | | | | 230 | 6000 (4000 – 8000) | 5250 (3500 – 7406) | 1.04 (0.93 – 1.16) | 0.46 |  |
|  | Frequency of breakfast use (days / week) | | | | | | 198 | 7.0 (5.0 – 7.0) | 7.0 (3.0 – 7.0) | 1.05 (0.92 – 1.19) | 0.50 |  |
|  | Vegetable intake (grams/day) | | | | | | 259 | 114 (86 – 157) | 100 (71 – 150) | 1.02 (0.97 – 1.07) | 0.50 |  |
|  | Fruit intake (grams/day) | | | | | | 259 | 100 (57 – 171) | 71 (29 – 171) | 0.99 (0.96 – 1.03) | 0.67 |  |
|  | Meeting recommendations vegetable intake ^5^ | | | | | | 259 | 36 / 208 (17.3) | 10 / 51 (19.6) | 0.86 (0.39 – 1.87) | 0.70 |  |
|  | Meeting recommendations fruit intake ^5^ | | | | | | 259 | 43 / 208 (20.7) | 10 / 51 (19.6) | 1.07 (0.50 – 2.30) | 0.87 |  |
|  | Meeting recommendations fruit and juice intake ^6^ | | | | | | 259 | 62 / 204 (30.4) | 11 / 51 (21.6) | 1.59 (0.76 – 3.30) | 0.22 |  |
|  | Meeting recommendations fruit- and vegetable intake ^5^ | | | | | | 255 | 16 / 203 (7.9) | 4 / 50 (8.0) | 0.98 (0.32 – 3.08) | 0.98 |  |
|  | Alcoholic beverages (units / day) | | | | | | 253 | 0 (0 – 0.2) | 0 (0 – 0.2) | 0.93 (0.48 – 1.83) | 0.84 |  |
|  | Total moderate to vigorous physical activity | | | | | | 238 | 330 (126 – 900) | 390 (120 – 950) | 1.00 (0.99 – 1.02) | 0.59 |  |
|  | Meeting recommendations guideline physical activity | | | | | | 254 | 147 / 202 (72.8) | 38 / 52 (73.1) | 0.99 (0.50 – 1.96) | 0.97 |  |
| *Partner characteristics* | Age (years) | | | | | | 289 | 32.9 (29.7 – 36.6) | 33.6 (29.6 – 38.2) | 0.99 (0.94 – 1.03) | 0.58 |  |
|  | BMI (kg/m^2^) | | | | | | 247 | 27.9 (24.5 – 31.7) | 26.5 (24.1 – 29.1) | 1.07 (1.00 – 1.14) | **0.04** |  |
|  | Non-Caucasian | | | | | | 288 | 18 / 225 (8.0) | 9 / 63 (14.3) | 0.52 (0.22 – 1.23) | 0.14 |  |
|  | Smoking | | | | | | 286 | 86 / 224 (38.4) | 31 / 62 (50.0) | 0.62 (0.35 – 1.10) | 0.10 |  |
| All determinants were assessed at baseline. Results of univariable logistic regression analyses on determinants of completion. ORs indicate odds for completion of the lifestyle intervention program as defined by not missing ≥2 consecutive sessions given the determinant is present. ^a^ Waist-hip ratio: per 0.10 increase. ^b^ Baseline calorie intake: per 100 kcal increase. ^c^ Baseline step counts: per 1000 steps increase. ^d^ Baseline vegetable and fruit intake: per 10 grams / day increase. ^e^ Total moderate to vigorous physical activity: per 30 min / week increase. ^1^ Socioeconomic status score (Netherlands Institute for Social Research (SCP)) in year 2010 relative to Dutch average of 0, a higher score represents a higher socioeconomic status. ^2^ Weight change during the first 1,5 months of the intervention program. ^3^ Calculated upon the highest and lowest weight in the past 10 years. ^4^ Measured by the Short-Form-36 Questionnaire. ^5^ Dutch Guidelines for a healthy diet 2006: minimal recommended fruit and vegetable intake 200 grams per day. ^6^ Dutch Guidelines for a healthy diet 2006: of minimal recommended fruit intake of 200 grams/day a maximum of 100 grams of fruit can be substituted by 1 glass of fruit juice daily. N.a.: not applicable. | | | | | | | | | | | | |
